# Using the Self-Management Assessment Scale for Screening Support Needs in Type 2 Diabetes: Qualitative Study

**DOI:** 10.2196/16318

**Published:** 2020-09-15

**Authors:** Ulrika Öberg, Carl Johan Orre, Åsa Hörnsten, Lena Jutterström, Ulf Isaksson

**Affiliations:** 1 Department of Nursing Umeå University Umeå Sweden; 2 Department of Computer Science and Media Technology DVMT, Malmö University Malmö Sweden

**Keywords:** eHealth, internet, type 2 diabetes, self-management, primary health care, qualitative research, nursing

## Abstract

**Background:**

Globally, most countries face a common challenge by moving toward a population-based structure with an increasing number of older people living with chronic conditions such as type 2 diabetes. This creates a considerable burden on health care services. The use of digital tools to tackle health care challenges established views on traditional nursing, based on face-to-face meetings. Self-management is considered a key component of chronic care and can be defined as management of the day-to-day impact of a condition, something that is often a lifelong task. The use of a screening instrument, such as the Self-Management Assessment Scale (SMASc), offers the potential to guide primary health care nurses into person-centered self-management support, which in turn can help people strengthen their empowerment and self-management capabilities. However, research on self-management screening instruments is sparse, and no research on nurses’ experiences using a digitalized scale for measuring patients’ needs for self-management support in primary health care settings has been found.

**Objective:**

This paper describes diabetes specialist nurses’ (DSNs) experiences of a pilot implementation of the SMASc instrument as the basis for person-centered digital self-management support.

**Methods:**

This qualitative study is based on observations and interviews analyzed using qualitative content analysis.

**Results:**

From the perspectives of DSNs, the SMASc instrument offers insights that contribute to strengthened self-management support for people with type 2 diabetes by providing a new way of thinking and acting on the patient’s term. Furthermore, the SMASc was seen as a screening instrument with good potential that embraces more than medical issues; it contributed to strengthening person-centered self-management support, and the instrument was considered to lead both parts, that is, DSNs and patients, to develop together through collaboration.

**Conclusions:**

Person-centered care is advocated as a model for good clinical practice; however, this is not always complied with. Screening instruments, such as the SMASc, may empower both nurses and patients with type 2 diabetes with more personalized care. Using a screening instrument in a patient meeting may also contribute to a role change in the work and practice of DSNs.

## Introduction

A need for structural changes in health care systems has emerged due to demographic changes and an increasing number of older people with chronic diseases such as type 2 diabetes (T2D), hypertension, chronic obstructive pulmonary disease, and asthma [1-3]. T2D is increasing in prevalence and constitutes a major cause of morbidity and mortality globally. In addition to contributing to a significant decline in health status in many patients, this condition creates considerable burden on health care services [2,4]. Self-management is considered a key component of chronic care and can be defined as the management of the day-to-day impact of a condition, which is often a lifelong task [5]. Self-management support and evaluation of patients’ self-management efforts are most often based on glycated hemoglobin (HbA_1c_) values and other measurements such as blood pressure and blood lipids. However, diabetes services are often unable to meet patients’ needs, such as emotional adjustment [6]. The struggle for people with T2D to manage self-care is therefore seldom evaluated [7]. However, most instruments focus on medical issues while instruments assessing patients’ perspectives and special needs for self-management support are lacking.

The development of digital tools for self-monitoring is rapidly increasing and becoming more common in chronic diseases. When implementing digital self-management support programs, there is a challenge to change from a traditional biomedical care approach based on monitoring and advice toward person-centered care based on empowerment [8]. Recent studies show that there are split opinions about using digital tools for self-management support among diabetes specialist nurses (DSNs) [9] and people with T2D [10]. Using digital tools challenges established views on traditional nursing based on face-to-face meetings and the importance of the care relationship in itself. In addition, patients seem to be quite positive about using eHealth for self-management support, while DSNs perceive that they lack an overview and are working in a digital chaos [9,10]. This study attempts to bridge this divide by applying the concept of person-centered self-management guidance using a screening instrument—the Self-Management Assessment Scale (*SMASc*) [11].

A broad definition of self-management implies both activities and support of chronic conditions, which may vary [12,13]. Self-management is defined as daily tasks that individuals must undertake to live comfortably with a chronic illness by gaining confidence in dealing with medical management, role management, and emotional management [10,12,14-16]. Self-management support, on the other hand, is defined as the provision of education and supportive interventions by health care professionals to increase patients’ skills and confidence in managing their health problems. This includes regular assessments of problems, goal setting, progress, and problem-solving support [16,17]. Emotional support for coping with the existential and emotional impact of having chronic conditions is seldom included in an annual visit to diabetes clinics [7].

The demands of governments and policy makers on the development of digital care are increasing. Digital care is suggested to decrease pressure on health services, but changes in responsibilities for patients and health professionals are not sufficiently evaluated or reported [18-21]. In Sweden and many other Western countries, DSNs—specialist nurses with education in diabetes care and working within primary health care—are the professional groups that most often meet and provide self-management support to people with T2D [22-24]. This study focuses on DSNs and their experiences of participating in a pilot implementation of person-centered self-management support for people with T2D, where digital tools are combined with the goals of person-centered care. This combination has the potential to enable tailored solutions and individual approaches that strengthen self-management capabilities, self-efficacy, and patient empowerment [9,25,26].

**Figure 1:**
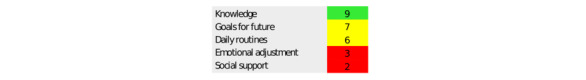
An example of one measurement and interpretation of the Self-Management Assessment Scale.

Within a pilot implementation, a self-assessed screening instrument (*SMASc*) was introduced that could assess the needs for self-management support. The *SMASc* is a short, validated screening instrument developed within our research group [11]. The instrument screens for strengths and possible barriers for self-management to be used in conversations between the person with T2D and the DSN. The *SMASc* instrument assesses 5 areas important for effective self-management over time: knowledge, goals for the future, daily routines, emotional adjustment, and social support, all generated from the literature on patient perspectives on chronic illness, including T2D and related self-management challenges [11], and it visualizes the results as an automatically generated profile (Figure 1). Each area of the *SMASc* has cutoffs between low value (red; acute need for self-management support), median value (yellow; no acute need for self-management support), and high value (green; no need for self-management support), directing the conversation regarding self-management support between the nurse and the patient [11].

The DSNs, when using the *SMASc* in the diabetes clinic, involved their patients in reflective conversations about the patients’ needs for self-management support in the various areas. They could also suggest other digital resources such as apps and websites for patients to improve self-management in problem areas. The *SMASc*, therefore, offers the potential to guide nurses into person-centered self-management support, which in the next step could increase people’s responsibility for their health and strengthen their empowerment and self-management capabilities [10].

Research on experiences using self-management screening instruments is sparse, as no published research on nurses’ experiences using digital scales for measuring patients’ needs for self-management support in primary health care settings has been found. To address this knowledge gap, it is important to gain more insight into and highlight this topic. Therefore, this study aims to describe DSNs’ experiences of a pilot implementation of the *SMASc* instrument as a basis for person-centered digital self-management support.

## Methods

### Design

This study is a part of a larger randomized intervention project (ClinicalTrials.gov [NCT03165084]) that aims to design and implement person-centered interactive self-management support in primary health care in the north of Sweden. More information about the project’s design, setting, and intervention is documented in a study protocol [27]. This study uses a qualitative descriptive approach based on participatory observations and individual interviews.

### Setting and Participants

This study was performed in 3 primary health care centers in Sweden, with 5 DSNs managing the diabetes clinics. DSNs are fundamental to this study because they introduce the *SMASc* instrument and, when needed, provide self-management support for people with T2D. The characteristics of the participating DSNs are given in Table 1.

**Table 1:** Demographics and clinical characteristics.

Characteristics		Participants (n=5), n (%)
**Gender**		
	Female	4 (80)
	Male	1 (20)
**Education level**		
	Primary health care nurse	5 (100)
**Age (years)**		
	<50	2 (40)
	≥50	3 (60)
**Years working in the current job**		
	≤5	2 (40)
	6-10	1 (20)
	≥11	2 (40)

### Preparation and Accomplishment of the Pilot Implementation

The participating DSNs were provided with a 1-day group training session, which included role-playing. In addition, they received a two-hour introductory session with the first author (UÖ). The training involved learning how to use the *SMASc* instrument to score and discuss the needs for self-management support and to develop person-centered plans for self-management support together with patients. The participating DSNs invited patients at their annual visit to their diabetes clinic and asked them to score their self-management support needs by using the *SMASc*. Thereafter, they were expected to discuss the 5 areas of the *SMASc*, with a particular focus on low-scoring areas. The profile of the *SMASc* results was expected to make the discussion more person-centered, and patients were also, when needed, recommended digital self-management support through the webpage [27] or the app, MySugr [28-33]. There was a handout manual containing instructions for DSNs about the *SMASc* and how to interpret the scoring and how to cope with possible barriers. The first author (UÖ) visited the intervention practice sessions on the first day of the intervention to provide additional support and to ensure that the DSN had understood the information and was able to work with the screening instrument in their consultations with the patients with T2D.

### Data Collection

Participatory observations of clinical visits (n=14) and following individual face-to-face interviews with each of the DSNs were conducted between September 2018 and February 2019. Participatory observations focused on the interaction between the DSN and the patient during consultations. The observer adopted an *observer as participant* approach, interacting only with participants if it was necessary to make a participant feel more at ease with the observation process [34,35]. Field notes were used while taking observations and were analyzed by identification and categorization of the types of interaction and further if there were any special occurrences observed during the visit. To ensure credibility and dependability, one author (ÅH) experienced in qualitative analysis confirmed the data and categorization. The observations were not audiotaped [35,36]. According to Silverman and Marvasti [37], the context of the observation is fundamental for the quality, further one has to be aware of facial expressions, gestures, and movements—all key data while making an observation. These types of observations were then used to interpret various situations and were used in the following interviews [37]. The interviews included open-ended questions, were audiotaped, and lasted for 60 to 90 min. On the basis of the observational data, a general semistructured interview guide was complemented with the questions. The opening question was, “Have you ever used any digital screening tool to measure the patient's need for self-management?” Examples of other questions included the following: “How is it to use the *SMASc* instrument in the patient meeting compared to before?” “Can the *SMASc* instrument highlight the needs of support that the patient is most in necessity of? “Would you be able to tell in what way?” The interview guide provided a flexible frame for questioning and domains (areas) covering topics about DSNs’ knowledge and perception in using the *SMASc* instrument for person-centered guidance and self-management support in their meeting with patients.

### Data Analysis

Qualitative content analysis, as described by Graneheim and Lundman [38,39], was used to analyze the interviews [40]. This is an appropriate method to highlight people’s thoughts about their experiences and their actions [38] and focuses on describing variations and identifying similarities and differences in the text by analyzing the manifest as well as the latent content. Themes at various levels were identified in this study. Subthemes are expressed closer to the interview texts, whereas the themes are expressed more on a latent level, that is, with a higher degree of interpretation [38,39].

The data material was transcribed verbatim by the first author (UÖ). The analysis was conducted in several steps. First, all transcribed text materials (also field notes from observations) were read thoroughly to assess the situation. Next, the text was divided into meaning units, answering the aim of this study. Each meaning unit was condensed and coded. During this process, the first author with help from coauthors continuously returned to the original text to ensure that the core meaning of the meaning units was maintained. This continuous cross-referencing process was maintained throughout the analysis. Similar codes were grouped into subthemes, which were later sorted and abstracted into themes. Finally, the latent meanings of the themes were interpreted and described as a main theme with a higher level of interpretation and abstraction [38,39]. During this procedure, to reach reliability, all authors discussed and reflected on the interpretation, sorting, and labeling of codes, subthemes, themes, and the main theme until consensus was reached.

### Ethical Considerations

The Regional Ethical Review Board at the Umeå University approved the study (Dnr 2014-179-31M), which was conducted according to the ethical principles described in the Helsinki Declaration [41]. All participants, including patients who participated in the observations, were informed about the study both in writing and verbally before giving their written informed consent. Transcripts were anonymized, and the participants were ensured confidentiality and were free to withdraw at any time. They were also informed that in case of any concerns, they could get their concerns clarified and any data collected could be excluded from the analysis, but none of the participants made such demands.

## Results

Participants’ experiences of the pilot implementation of the *SMASc* instrument in primary health care were mostly positive. DSNs expressed various feelings, and from their narratives, 4 identified themes describing their experiences of using the *SMASc* instrument were identified and labeled: *A screening instrument with good potential*, *Embraces more than medical issues*, *Strengthen person-centered self-management support*, and *Both parts develop through collaboration*. The main theme that tied the 4 themes together was formulated—*A new way of thinking and acting on patients’ terms.* To increase the transparency of the interpretation, themes and subthemes are illustrated with quotations. An overview of the results is shown in Textbox 1.

**The main theme, themes, and subthemes emerging from the analysis.**

Main theme: A new way of thinking and acting on patients’ terms

Themes and subthemes:

1. A screening instrument with good potential
  - An educational, easy-to-use tool
  - Builds on honest answering
2. Embraces more than medical issues
  - The patient becomes more than the disease
  - A door opener to address difficult topics
3. Strengthen person-centered self-management support
  - Conditions for tailored counselling satisfying
  - Patients become more empowered
4. Both parts develop through collaboration
  - Incentives for self-management and support
  - An opportunity for reflections and reframing

### A New Way of Thinking and Acting on the Patient’s Term

The use of the self-reported *SMASc* instrument to screen and estimate the patient’s needs or levels of self-management support in the daily work in the diabetic clinics involved various feelings among the participants. The DSNs emphasized that they found that using the *SMASc* in their meeting with the patient with T2D involved having a more in-depth conversation between them. They expressed that using the instrument, which embraced more than medical issues, led to more person-centered support and further that it was built on mutual trust developed through collaboration. This was interpreted as *A new way of thinking and acting on patient’s terms—*the DSNs received a new tool that altered their way of approaching the patients.

#### A Screening Instrument With Good Potential

The participants described the *SMASc* as an easy-to-use tool to use for in-depth communication with their patients, but a prerequisite was that the patients should respond honestly when scoring using the *SMASc*. Therefore, the *SMASc* was interpreted to be a potentially good instrument for use in DSNs’ daily work with patients with T2D.

##### An Educational, Easy-to-Use Tool

The DSNs described that they had experienced the *SMASc* to have streamlined the meeting with the patient. The DSNs also expressed that no extra time was needed to fit the *SMASc* into their regular workflow. The tool was also perceived as easy to fill in and educational for both the patient and the nurse. It took approximately 1 to 2 min to fill in on an iPad, and they received the scoring directly. Furthermore, it was also easy to interpret the results, with scoring points and the demonstrative colors—red, yellow, and green. By interpreting the scoring and demonstrating it for the patient *on the table*, the DSNs expressed that it was a helpful way to address important topics that could be meaningful for the patient:

...the questionnaire was easy for them to fill in...it became easy to discuss the results [of SMASc]...with all the colors...like the traffic lights...Together [DSN and patient] then we decided what topic we should concentrate on...

##### Builds on Honest Answering

The prerequisites of using a tool such as the *SMASc* are that the patients’ answers must be based on truth and honesty; otherwise, it fills no function. The DSNs verbalized that they had experienced that *for some reason, a few of the patients probably did not answer honestly on the* *SMASc*. For example, if a patient had their partner present during the visit and the patient scored high on *social support* but the DSN simultaneously knew that the last time the patient visited, he or she had complained about his or her spouse’s lack of understanding:

...for example, if a patient brings his partner to the visit to the diabetic clinic...then it may become difficult for them to fill in SMASc truthfully about having poor social support...then it will be incorrect...and it can then be difficult for me to talk to the patient about it...it can be sensitive...

#### Embraces More Than Medical Issues

The DSNs described that they usually checked the patients’ laboratory values and body weight to judge how the patients *behaved*. They had tried to educate the patients, foremost in pathophysiological and medical topics if the values deviated from the normal. Psychosocial topics were also important, but these had been difficult to address before. The participants also expressed that from their point of view, effective self-management is often dependent on the collaboration between the patient and the DSNs. Although the patient was viewed as a person and thereby more than only a disease, the focus during visits was often on measurements, but by using the *SMASc* and the conversation around it, the DSNs gained a better understanding of the patient’s overall life situation, thereby increasing his or her empathetic understanding. The *SMASc*, which embraces more than medical issues, could highlight topics that have seldom been discussed earlier.

##### The Patient Becomes More Than the Disease

The *SMASc* conversation focused on the following topics: patients’ knowledge, goals for the future, daily routines, emotional adjustment, and social support. During the annual visits, the nurses had to follow up the standardized annual medical measurements at the same time. The DSNs stated that patients’ daily decisions had a huge impact on their health, and they must therefore be active and informed about their medical issues. They advocated that measurements such as HbA_1c_ and other curves are important but could feel bad when such values were normal, and they did not listen to the patients’ other struggles. The *SMASc* helped them to understand and make visible which area the patient was struggling with. The DSNs expressed how they were discussing problem areas chosen by the patient. By focusing on the patients’ everyday life priorities, communication was strengthened, and they got a better understanding of the patient’s overall situation:

I think it [results of SMASc] contributes to being able to meet the patient where he or she is...and I think I listen more, actually...it is easier to understand the patient's needs because the questionnaire covers wide areas...

##### A Door Opener to Address Difficult Topics

The DSNs described it as a door opener in communication when the patient obtained the results from the *SMASc* and it became clear what topics should be raised in the conversation.

The DSNs also felt that it enabled them to provide better care. The use of *SMASc* was described as a new way of thinking and working. Areas that received low points were those that the DSN concentrated on in the conversation with the patient. On the basis of the answers in the *SMASc*, the DSN could better assess where to start and what to focus on in the discussion. This provided a foundation for new directions in their self-management support and answers on what both patients and DSNs should continue with. If patients scored highly on *knowledge*, the DSNs would realize that they need not repeat information about things that the patients understood:

...by using the questionnaire involves a new way of thinking and working...it guides both me and the patient on what to concentrate on in our conversation...

#### Strengthening Person-Centered Self-Management Support

The DSNs expressed that by using the *SMASc*, it became clear and visible for them if a patient scored low in any area. This allowed the DSN to approach the patient in the conversation through an in-depth dialogue to better address the patient’s personal needs. In this way, the DSNs gained insight into what to focus on, and together with the patient, they could set up an individual care plan and support them accordingly. Thus, these individual strategies led to tailored and person-centered self-management support.

##### Conditions for Tailored Counseling Satisfying

The DSNs described that the *SMASc* gave them the opportunity to address personal issues about what motivates patients in their self-management. On the basis of patients’ scores and the following discussion, a range of topics that have been discussed previously, such as personal risk factors, readiness for change, patients’ self-management needs, preferences, and health behaviors, were also highlighted. The DSNs experienced that it became easier to develop an individualized self-management plan together with the patient after the *SMASc* scoring. The DSNs described that although they had only used the *SMASc* for a short time, they considered that the instrument provided them and the patient with the prerequisites for more person-centered care and tailored coaching. Subsequently, they believed that it strengthened *the patients’ ability to carry out self-management activities. The DSN reported that it sometimes took time for them to build a trustworthy relationship with patients with T2D, but by using the* *SMASc*, it became easier for them to come closer to patients and to discuss sensitive topics. They stated that conversations were considered to improve compared with earlier conversations:

...some things that are addressed in the questionnaire may be,...like different type of goals or needs for support for future plans...that may not always come up at the usual visits to the diabetic clinic..., now it will be easier to approach such topics as well...you want to be able to reflect on what has been done and what effects that it has resulted in...

##### Patients Become More Empowered

The DSNs reported that their discussions with patients about the low-scoring areas in the *SMASc* motivated the patients to more effectively improve self-management. By making this color scoring visible and sharing between them, the patients seemed to cope better and gain restored strength to perform new activities or better adapt to situations. However, the DSNs all agreed that this required an involved patient and that not all patients could easily be reached. Within the project, DSNs were instructed to refer patients to digital sources for self-help. Some patients seemed to have a positive reaction to this, and the option to monitor themselves without the nurse’s involvement was viewed as positive and surprising for the nurses. Using a digital tool, such as the *SMASc*, was perceived as a new solution for some of their patients, and the DSNs saw that they would have a new task as coaches in digital self-management support instead of educators:

...one might also think that it [results of SMASc] also can motivate the patient to take responsibility for their own health care...the insights and transparency are not only for me but also for the patient...time for self-reflection...

#### Both Parts Develop Through Collaboration

Using the results of patients’ *SMASc* scores helped both DSNs and patients to understand more about themselves and one another. This provided an opportunity for reflection and evaluation from earlier discussions. This was suggested by the DSNs to help the patients strive toward more effective self-management and help the DSNs to understand what type of support they could provide to the patient. Therefore*,* the *SMASc* was useful for both parts and guided them toward better collaboration and understanding as well as development for self-management and support.

##### Incentives for Self-Management and Support

DSNs highlighted that the *SMASc* gave them incentives for better and more person-centered support and that topics became visible to patients they had not thought of before, such as future goals. The DSNs described that it was this collaborative approach that helped the patients to acquire skills and confidence to manage their condition. Participants also highlighted the need for new self-management strategies and allowed nurses and patients to make a personalized assessment of problems. The DSN expressed that patients with T2D could receive a new kind of support such as emotional support or guidance on illness integration, including issues that are most important to them at this point of departure:

...what is important is that the patient has good illness integration...that everyday life should be the most important, living with the disease [T2D] is challenging...but, it should not take over your whole life..., it should not feel like a mountain that one can’t climb...

##### Opportunities for Reflections and Reframing

The DSNs described that they thought that the *SMASc* could be a useful tool in their conversations with patients as it provided an opportunity for reflection, evaluation, or feedback on the previous efforts for both patients and DSNs. It provided answers to what is less good in health care and what changes had to be made in the patient’s own treatment plan. One DSN explained it as having a new mirror image, which included things that had already been done and even reflected on how the self-management support had been perceived. This gave both patients and DSNs an opportunity for reframing and changing the direction forward. The DSNs expressed that the annual assessment with the *SMASc* to obtain a receipt on how the patient managed his or her life with diabetes must be implemented in full scale:

...what I think about this SMASc...it can be of help...new way to work...no question about that...if you [the patient] fill it in...then I, as a diabetes nurse get the feedback from the patient...in the results of SMASc...about previous efforts, about my efforts on the patient's efforts...

## Discussion

This paper aims to describe DSNs’ experiences of a pilot implementation of the *SMASc* instrument as a basis for person-centered digital self-management support. To understand the experience of using such an instrument and what it means for a day-to-day practice in the setting of a diabetes clinic, the use of such digital resources such as the *SMASc* needs to be studied. The central focus in such an observation is the interaction between patients and nurses. Combined with interviews where the DSNs could directly describe and explain their experiences, this study captures a reflexive account of using the *SMASc*. The main theme concluded that *the use of SMASc* involved *a new way of thinking and acting on patients’ terms*.

The DSNs highlighted that the *SMASc* instrument was perceived as *a screening instrument with good potential* to facilitate discussions of self-management strategies and thoughts around new ways of thinking and acting on patients’ terms in the health care situation. The analysis also indicates that the use of *SMASc* as a digital screening tool offers the possibility of another kind of patient meeting where *both parties are developed through collaboration.* As is seen with other examples of using digital resources, applications are altered and reformed through utilization in everyday practices [42-44].

In this study, the DSNs expressed that using the *SMASc* helped them to restructure the consultation method. They realized that the *SMASc* had the potential to help them focus on matters of relevance for the patient, thereby enabling them to learn more about the patients’ needs. It was expressed as positive that the *SMASc embraces more than medical issues* and *strengthens person-centered self-management support*.

The *SMASc* offered an overview that helped the DSNs to change focus and highlight issues of importance for the patients, aspects that might otherwise not have been mentioned if they had used their former daily routines.

The *SMASc* can make it possible to identify the barriers to self-management, and the DSN is given a resource to assess patients’ self-management needs. This is in line with other related studies and initiatives [45-49] aimed to support nurses with analytic tools to better understand patients’ situations and to evaluate self-management interventions. One such example is the development of the Self-Management Screening (SeMaS), by Eikelenboom et al [50,51], a SeMaS tool aimed to support the creation of patient profiles that could support nurses in counseling and the evaluation of self-management interventions in primary care. First, an important difference between SeMaS and *SMASc* is the length. SeMaS includes 27 items and the *SMASc* includes 10 items, in favor of *SMASc* in clinical practice. Second, SeMaS is based on psychological theories related to behavior change and internet use, whereas the *SMASc* is developed inductively from *the experiences of patients living with T2D. SeMaS is not translated into Swedish, and we had no knowledge of the instrument when we started to develop the SMASc* [11,50].

The *SMASc* provides a visual response that helps the DSNs to prioritize and provide attention and support to areas of importance. Other topics on the DSN agenda could therefore wait and be managed later or even disregarded. In the context of Swedish primary care, the DSN meets with the patient annually. As consultation traditionally follows a *one-size-fits-all* [52] kind of a character, thereby giving a clear concept about what to expect and what questions should be asked, the *SMASc* provides an opportunity for an alternative person-centered approach. With this background, the DSNs in this study discovered that the *SMASc* gave them an incentive and support to find out more about how patients experienced their situation and options for self-management. It specifically focused on what patients found to be relevant to discuss and what they did not want to bring up, which did not always comply with the topics the DSN routinely chose to pay attention to. Interestingly, although person-centered care has been promoted for several years [53-57], the DSNs expressed that it is difficult to discuss personal topics such as social support, emotional adjustment, and goals for future without a *manual* like the *SMASc*. However, the conversational space through the *SMASc* suddenly became wider, which led to counseling that could go beyond questions about medication and the importance of compliance in routines.

The *SMASc* was experienced as easy to use, and the DSNs highlighted that they appreciated that the *SMASc* also gave them important suggestions, thereby helping and allowing them to address the issues that they would have otherwise forgotten or left out. Technological development in health care has been described as living in a digital chaos [9]. The development of digital resources that includes one’s perspective seems to prolong engagement in it. This is supported by other related studies concerning patients’ engagement in digital resources. Lupton [58] describes that patients will only use new technologies if they are relevant to their problems and are engaging, easy to use, and effective in achieving change. The DSNs in this study found this tool to be both engaging and easy to use as well as effective in achieving change. The use of *SMASc* provides a structure for personalized counseling. The design of *SMASc* was pragmatic; the aim was to offer a screening tool that is easy to use and easy to interpret. The visual result is documented as a traffic light screenshot—something that can be attached to patients’ health records in the future. The digital prototype presented to the DSNs in this study had a low key visual design, as the prime focus was to explore how the result of the instrument was utilized in the meeting by both DSNs and patients. The color scheme—green, yellow, and red—however, follows a traffic light metaphor [59,60], where the latter two colors guide the DSN toward the topics that need attention. This metaphor was perceived to be pedagogic by the DSNs, even if the design can be improved.

Using the *SMASc* was important for the DSNs as it helped them to discover a new way of thinking and acting on patients’ terms. The use of screening tools *such as the* *SMASc* will imply a change of roles in the DSN work models and practices. The emerging challenge here concerns the structure of the meeting with the patient and the strategies by which the DSNs moderate the conversations in a person-centered manner. The DSNs experienced the *SMASc* as a resource that functioned as a facilitator and initiator in their interaction with the patients about sensitive topics. It facilitated the conversation between them, and it was felt that it led to shared engagement concerning self-management and support needs.

### Strengths and Limitations

A strength of this study is the qualitative design that allows for an understanding of DSN experience based on the actual use of the *SMASc* instrument. Its closeness to daily DSN practice is another strength that emerged, as the pilot implementation was integrated into daily processes as much as possible. This enhances its relevance to decision makers as an application in daily practice that is proven feasible. Furthermore, the sample of participating nurses and the number of observations were considered sufficient. However, some limitations of this study need to be discussed. Some challenges existed in the recruitment phase, where 2 DSNs were recruited later than the others. The DSNs in this study had volunteered to participate and had a specific interest in diabetes care and may have tended to express more positive opinions than average. Therefore, our findings do not necessarily reflect the perceptions of other DSNs.

### Conclusions

To our knowledge, this is the first study that has reported DSNs’ experiences of using a screening instrument to measure the level of needs of self-management support for patients with T2D. The results indicate that from the perspective of DSNs, the pilot implementation of the *SMASc* instrument offers insights that contribute to strengthened self-management support for people with T2D and serves as a guide to person-centered care in clinical practice. However, to obtain this, the benefits rely on whether nurse-led digital self-management support is prioritized in the organization and whether the DSNs are engaged in person-centered care in practice during the visit.

It is important to understand that the implementation of an instrument such as the *SMASc* may also challenge the traditional roles of DSNs. Even though person-centered care is advocated as a model for good clinical practice, this is not always complied. Instruments such as the *SMASc* may contribute to making such a shift happen. This study shows that DSNs experienced the use of *SMASc* as an enhancement to diabetes nursing and that it has the potential to improve self-management among patients with T2D. This study supports that the *SMASc* is ready to be used but some minor technical refinement and design improvements may need to be done before full-scale implementation.

## Abbreviations

DSN: diabetes specialist nurse
HbA_1c_: glycated hemoglobin
SeMaS: Self-Management Screening
SMASc: Self-Management Assessment Scale
T2D: type 2 diabetes
